# Communicating science: epigenetics in the spotlight

**DOI:** 10.1093/eep/dvaa015

**Published:** 2020-11-18

**Authors:** Stephanie O M Dyke, Catherine A Ennis, Yann Joly, Jörn Walter, Reiner Siebert, Tomi Pastinen

**Affiliations:** d1 Faculty of Medicine, Centre of Genomics and Policy, McGill University, Montreal (Quebec), H3A 0G1, Canada; d2 Human Early Learning Partnership, BC Children's Hospital Research Institute, University of British Columbia, Vancouver, BC, V5Z 4H4, Canada; d3 Saarland University, 66123, Saarbrücken, Germany; d4 Institute of Human Genetics, Ulm University Medical Center, Ulm University, Albert-Einstein-Allee 29, D-89081, Ulm, Germany; d5 Department of Human Genetics, McGill University and Genome Quebec Innovation Centre, Montreal (Quebec), H3A 0G1, Canada; d6 Center for Pediatric Genomic Medicine, Children’s Mercy Kansas City, Kansas City, MO 64108, USA; d7 Faculty of Medicine, Montreal Neurological Institute, McGill Centre for Integrative Neuroscience, McGill University, Montréal, Quebec, H3A 2B4, Canada; d8 Amphoraxe Life Sciences, Inc., Vancouver, BC, V6L 3C9, Canada

**Keywords:** epigenetics, science communication, public understanding of science

## Abstract

Given the public interest in epigenetic science, this study aimed to better understand media representations of epigenetics in national newspaper coverage in various regions in North America, Europe, and Asia. Content analysis was used to study media messages about epigenetics, their policy focus, and the balance of the reporting. We identified several recurring themes in the news reports, including policy messages relating to individual and societal responsibilities. We also found shortcomings in the media’s portrayal of epigenetic science, and sought to identify potential causes by considering the underlying scientific evidence that the media reported on. A case study analysis showed that the results of epigenetic studies were often overstated in academic research publications due to common experimental limitations. We suggest that defining standardized criteria with which to evaluate epigenetic studies could help to overcome some of the challenges inherent in translating complex epigenetic research findings for non-technical audiences, and present a Press Kit template that researchers can adapt and use to aid in the development of accurate and balanced press releases.

## Introduction

Epigenomics, the study of epigenetic mechanisms across the entire genome, is shedding light on how interactions with the environment lead to changes in gene expression, some of which might affect individual susceptibility to a range of diseases [1]. Many aspects of the field are of interest to non-academic audiences. Epigenetics is widely seen as redefining our views of genetics, with particular relevance for popular perceptions of genetic determinism and heredity. Certain research areas, such as the social epigenetics of historical trauma, have resulted in ‘intense public appropriation’ [2]. Second, the potential for dietary and other lifestyle interventions to mitigate epigenetic risk factors has led to a proliferation of epigenetics-based health offerings, including everything from “epigenetic” shampoo and dietary supplements to yoga classes and life coaching. Direct-to-consumer DNA methylation tests for biological age [the ‘epigenetic clock’ (3, 4)] and male infertility are also starting to appear on the market.

As with any technical field, mass media play an important role in defining the public discourse about epigenetics [5], and there have been calls to study the content of epigenetics risk messaging [6–8]. We therefore sought to investigate media representations of epigenetics in national news coverage in various regions in North America, Europe, and Asia that are involved in the International Human Epigenome Consortium (IHEC), of which we are members.

IHEC was founded in 2010 with the primary goal of producing and disseminating high-resolution reference human epigenome maps for normal and disease cell types [9]. Alongside these sequencing and analysis efforts, several international working groups were tasked with complementary activities such as developing standards and guidelines. Of relevance to this study, the multidisciplinary Bioethics Working Group focuses on the relationship among epigenetic science, ethics, and policy, while the Communications Working Group's mandate encompasses internal and external communications, including public outreach. To support the efforts of both working groups, and to contribute to the wider discussion of how working scientists can best contribute to the public discourse about epigenetics, this study aimed to better understand how epigenetics is portrayed in the media to support the development of knowledge translation strategies, as well as to contribute to discussions of the ethical, legal, and social issues concerning epigenetics.

## Methods

### Article Selection

Countries and regions were selected according to IHEC member projects’ locations, and opportunities for collaboration and assistance with the identification of suitable media outlets and stories for translation and analysis. The study therefore included newspaper reporting on epigenetics in Canada, the USA, the UK, France, Germany, Hong Kong, Singapore, South Korea, and Japan.

The following academic databases were used to find national news coverage (excluding opinion pieces) in English or French for the period from July 2014–July 2015 [search term (of full text): epigenetic(s) or épigénétique]: ProQuest Newsstand, Canadian Newsstand Complete, Newscan.com (French language press). Newspaper website archives with English or French content were also searched directly with the same search terms epigenetic(s) or épigénétique (*Le Monde*, *South China Morning Post*, *The Korea Times*, *The Japan Times*). News stories that did not primarily focus on epigenetics were excluded. An additional five stories meeting selection criteria were received from collaborators who speak other languages (German) or with access to local listings of epigenetics media coverage. In total, 16 news articles, including feature articles, met the selection criteria for the analysis (Canada, 2; Germany, 2; Hong Kong, 2; Japan, 2; UK, 2; USA, 5; Singapore, 1). No news reports on epigenetics were found in the French national press for the period studied (July 2014–15), nor in the English-language press we considered from South Korea. Selected news articles in German were translated into English. Epigenetics news coverage (in Canada and the USA) was further assessed for the later period of January 2019–20, including local newspaper stories as well as national press (using the same search and inclusion criteria, and Canadian Newsstream, US Newsstream, and Nexis Uni databases).

### Media Content Analysis

Content analysis is a flexible technique that is particularly well suited to media studies as ‘the primary message-centered methodology’ [10, 11]. Qualitative and quantitative content analysis methods were used for this study, which included both inductive and deductive items in its coding framework. We focused on an analysis of the main messages that the media presented about epigenetics, and in particular, on determining the policy messages about epigenetics that were conveyed by the press. We also sought to assess the balance of the reporting in the news reports. The coding frame included the following: (i) main message(s) of the story; (ii) research evidence reported (including research ‘type’ and model organism used); (iii) supporting research authority (e.g. scientist’s comments on the research or field); (iv) reporting of uncertainty or research limitations; and (v) policy message(s). Policy messages were then categorized into themes (e.g. parental responsibility, public health, research investment).

### Case Study

We traced the research evidence referred to in the selected news reports to their original sources (academic scientific journal publications) in a case study of the stories with policy messages focused on parental responsibility (from 2014 to 2015). Our aim with this case study was to understand how some of the common distortions and overstatements regarding epigenetics were likely to have arisen. The scientific and media descriptions of the reported scientific research, the latter considered in both isolation and the context of the overall media story, were scored on a four-point scale (understated, fair, a little overstated, and very overstated). Scientific reports were scored by an epigenetics expert and the reasons for any overstatement were recorded. Media stories were scored by a science communication and policy expert. Media descriptions of the scientific research (in isolation) were scored separately by both experts (11/14 concordant scores; all three discordant scores were easily resolved upon re-examination).

## Results and Discussion

### Media Messages and Policy Emphasis

There were a total of 16 national news articles that met selection criteria in the period from July 2014 to 2015, including stories from most of the regions that we considered, indicating that the international mass media saw epigenetics as an important emerging area of science.

We considered the type of research that was included in the media reports selected for our study, that is, the focus of the studies that were reported on (e.g. transgenerational epigenetic effects), and the model organism used for the research. Most studies referred to by the press were either research into transgenerational and intergenerational epigenetic effects (*n* = 24) or acquired epigenetic effects over one’s lifetime (*n* = 15) (see Table 1). Research into *in utero* epigenetic effects affecting later health (*n* = 2) and the potential for epigenetics-based pharmaceuticals (*n* = 5) or diagnostics (*n* = 5) was less prevalent. There were also stories about epigenome mapping referring to the release of publications from the NIH Roadmap Epigenomics Mapping Consortium, an IHEC member project (*n* = 3). Of the research studies reported on, 33 used human samples and 17 used rodent models, suggesting potentially greater media interest in studies in humans. Due to language barriers and the geographic limitations of news article databases, the news pieces included in our study do not represent a complete sample of all national press coverage of epigenetics in several of the regions we considered, nor can we exclude selection bias for articles from those regions where English- or French-language academic news databases were not available.

**Table 1:** research of interest to the press

Research focus/model	Total	Human	Monkey	Rat	Mouse	Animal (not specified)	Plant
Transgenerational and intergenerational effects	24	11		3	8	1	1
Lifetime effects	15	10	1	2	2		
*In utero* effects	2	2					
Epigenetic drugs	5	3			1	1	–
Epigenetic diagnostics	5	4			1		
Epigenome mapping research	3	3					
Total	54	33	1	5	12	2	1

Research focus and model organism used for the research studies referred to by the press in the news reporting for 2014–15 (*n* = number of research studies, some of which might be referred to in several news stories and therefore counted multiple times).

The news stories consistently reported messages related to two main themes: (i) that epigenetics is transforming our understanding of the nature versus nurture debate; and (ii) that harmful epigenetic changes (for example, in response to stress) can be inherited, with the vast majority of the stories focused on transgenerational inheritance. Many factors were reported as causing epigenetic change, including nutrition, exercise, body mass, smoking, nicotine, alcohol, behavior, stress, pre-diabetes, autism, hormone treatment, and environmental pollutants. Media reports of the consequences of epigenetic changes in offspring included their potential role in physical fitness, obesity, cancer, pre-diabetes, diabetes, allergies, asthma, cardiovascular disease, autism, depression, and behavior.

Readers are receiving strong messages from the media about the “fatality” of epigenetic harm that is passed on through the generations, with serious impact on many areas of health, both physical and mental. This rather deterministic view is interesting given evidence that epigenetic effects are potentially malleable and at the very least dynamic in the context of much of the research reported (e.g. impacts of nutrition, behavior, life-style and exercise). It is also interesting in light of the ubiquitous media message that epigenetics is redefining the relationship between nature and nurture—in other words, that our genetics are *not* set in stone.

A similar dichotomy was seen in an analysis of metaphors used in media descriptions of epigenetics, which found that the metaphorical expressions used to discuss epigenetics were more dynamic than those used for genetics (such as ‘switching’ and ‘marking’ rather than ‘book’ or ‘code’), but also the use of fatalistic metaphors such as ‘curse’, ‘doom’, ‘poison’, and ‘time bomb’ [12]. Another study of British and American radio coverage of epigenetics found it to be couched in language as deterministic as for genetics research [13]. While social scientists have themselves put forth social representations of epigenetics strongly rooted in a breakdown of the boundaries between the biological and the social, and even hailed the ‘death’ of genetic determinism [14], they have also forewarned of the pernicious aspects of ‘epigenetic reductionism’ [15–17]. Referring to Jörg Niewöhner’s notion of the ‘embedded body’ from his ethnographic observation of environmental epigenetics research practices [18], Margaret Lock noted ‘the tendency, visible already in epigenetic research, to move rapidly toward systematized somatic reductionism’ [16].

On the other hand, in an analysis focusing mainly on German media, and on a broad range of publication types, the theme of how epigenetics is redefining the nature versus nurture debate was seen to have only gained media importance in more recent accounts, and of having a more positive slant in weekly magazine reports of an advice-giving nature. The authors report that these stories emphasize ‘an individual’s fixedly determined fate is not that “inevitable”, after all, but—as it is suggested—can be prevented by way of positively changing one’s own environment’ [19], a message of power and control that has also been found to be emphasized in marketing materials for epigenetic products and services, such as skincare creams and fitness and wellness programs [14]. Similarly, a recent study of a very broad range of media, including social media and commercial website publications, found that epigenetics was ‘steadily depicted as giving the general public the capability to “take control”’ [20].

In the news reporting from our study, we observe that publics around the world are being pushed to consider their personal as well as societal responsibilities in preventing epigenetic harm. The main policy messages found throughout the news reports could be loosely grouped under the themes of ‘parental responsibility’ (in *n* = 7 articles), ‘individual responsibility’ (*n* = 2), ‘social justice’ (*n* = 1), ‘public health’ (*n* = 1), ‘therapeutic potential’ (*n* = 4), and ‘research investment’ (*n* = 2) (see Table 2). Perhaps surprisingly, the potential for epigenetics-based pharmaceuticals and other epigenetic therapies was only mentioned in four of the news reports. The overarching focus was on preventable epigenetic harm.

**Table 2:** policy implications referred to in the news stories (2014–15)

Art.	Policy message	Theme
A1	Blame for impact of behavior on offspring	Parental responsibility
	‘Important addition to the ongoing debate about the health risks and regulation of new smokeless e-cigarettes’	Public Health (regulation)
	Argues researchers should seek to develop epigenome drugs targeting the same epigenetic switches flipped by diet and exercise	Therapeutic potential
A2	Need to be ‘extremely careful’ regarding the potential for blaming mothers	Parental responsibility
	‘Only mothers have the power to change the evolution of obesity’	Parental responsibility
B2	‘… suggest a vicious multigenerational cycle’; the new studies show that maltreatment, more prevalent in poor families, ‘damages children and perhaps even their children’s children at the most fundamental biological level’	Parental responsibility Social justice
B3	‘Through endurance training—a lifestyle change that is easily available and doesn’t cost much money, we can induce changes that affect how we use our genes and, through that, get healthier and more functional muscles that ultimately improve our quality of life’	Individual responsibility
C1	‘Parents could suddenly find themselves responsible for passing on not only their poor genes, but also their poor lifestyles’	Parental responsibility
E1	Mothers are responsible for the future health of children and future generations (code of conduct getting longer; surrogates will need contracts about what they can/cannot do)	Parental responsibility
E3	Though it might not be possible to reverse damage, good nutrition could spare offspring from other diseases	Parental responsibility
F1	‘If the DNA encoding could be corrected, would this be enough to make the men behave better?’	Therapeutic potential
	‘You may be able to make positive epigenetic changes, and do so today’	Individual responsibility
H1	‘Research into gastric cancer is neglected in the West, where the incidence of such cancers is lower. “Stomach cancer occurs more in Asia and we are well-placed to apply this technique in the region”’	Research investment Therapeutic potential
I2	‘The only way you can deliver on the promise of precision medicine is by including the epigenome’	Research investment Therapeutic potential

Along with responsibility toward future generations, the consequences that epigenetics might have for parenthood and its associated duties was a major theme—but with varying emphases, some stories “blaming” and others stressing the importance of *not* blaming parents and guardians (and some presenting both viewpoints). An example from the Canadian press:

‘Mothers in many subpopulations have evolved past a “metabolic tipping point” that makes obesity and poor physical fitness almost inevitable for their children and their children’s children’. [21]

Media attention to parents’ experiences and responsibilities, especially those of mothers, has previously been observed in epigenetics news coverage [22, 23]. Richardson *et al.* [23] have urged ‘researchers, press officers and journalists to consider the ramifications of irresponsible discussion’ in this area, pointing to the ‘long history of society blaming mothers for the ill health of their children’.

We followed up this analysis with a sample of news reports from Canada and the USA 5 years later (January 2019–20), to assess whether the content of epigenetics news reports had evolved in that time. National media interest in epigenetics did not appear to have grown as might have been expected from previous reports that epigenetics stories in the English-language press had risen from only a handful of reports annually in the early 2000s, to 481 articles in 2013 [12]. The Canadian national press only published one news story about epigenetics during the later period, and there were only two national stories in the USA, compared with 2 and 5 respectively during the earlier analysis period. It has since been shown that the year 2014 represented an inflection point in the diversification of both study topics in epigenetics as well as the range of historical events associated with trauma epigenetics in various media [2], so the earlier period may therefore have been a peak in national news coverage. However, we do not expect press interest in epigenetics to disappear anytime soon, and also included epigenetics news stories from the local press in our analysis for the later period (2 in Canada, 5 in the USA).

We found a greater focus on epigenetics applications (including diagnostics, treatments, and even insurance), as well as a different policy emphasis, in the later reporting. The main policy theme was ‘therapeutic potential’, of either epigenetic diagnostics or treatments (*n* = 5). Notably, there were much fewer policy messages about ‘parental responsibility’ (*n* = 2) than previously, although harmful *in utero* epigenetic effects were the focus of three stories, with one also referring to research into the effects of high-fat diets on sperm. We found similar messages about ‘research investment’ as previously (*n* = 2), and reports of the epigenetic effects of environmental toxins in messages related to ‘public health’ (*n* = 2). A new policy theme was that of ‘conflicts of interest’ in epigenetics research, from a report on the potential effects of a herbicide on future generations (along with the theme of ‘public health’). Another policy theme we had not seen previously was ‘insurance potential’ from a local US report about a company developing epigenetics-based insurance policies. Even more markedly than during the earlier period, the vast majority of studies referred to by the press continued to be human studies (17 human, 4 rat). Finally, consistent with the change in media focus, there was somewhat less attention paid to studies about inter- or trans-generational epigenetic inheritance (6/20 references to research results).

Our analysis lends further weight to emerging academic discussions about the potential of epigenetics science communication to both frame and potentially misguide public policy developments at a very early stage of its development [24]. The news coverage we considered did not generally appear to amplify dystopian views of epigenetic discrimination and new forms of eugenics [8]. However, the press is already widely drawing attention to potential policy implications that warrant careful examination. Furthermore, while we found a few examples of outright hyping, overall, the risk messaging seemed to be mostly skewed by overstatement of our current understanding of epigenetic changes, with regards to both their causes and their effects. This was especially striking in relation to the potential heritability of epigenetic changes, which was a key message of most news stories in the earlier reporting of the “new” field of epigenetics. We therefore sought to assess this phenomenon in more detail in both the media reporting and the scientific literature.

### Balance of the Reporting

Although reasonably good reporting practice was seen in many of the 2014–15 stories with respect to providing a balanced range of views on the subject, many news reports (9/16) did not emphasize the current lack of evidence or understanding in epigenetics research (see Table 3). We also found examples of accurate reporting on caveats and uncertainties: US stories reporting on the Roadmap project’s publications referred to the limitations of animal models and the ‘long road ahead’ to understanding epigenetics [25, 26]; a Canadian report focused on the message that the research was controversial and not supported by all experts [21]; and a story from Hong Kong included important caveats:

‘But there are controversies, notably regarding a study that found pesticide and fungicide impacts on pregnant rats led to changes that persisted for at least four generations—results that have proven hard to replicate’.

‘As yet, the results are based on a small sample size’. [27]

**Table 3:** balance of the 2014–15 news reports in different countries or regions

Country/ region	Balanced (overall)	‘Moderating’ views or evidence
Canada	2/2	– Refers to overhyping – ‘But that goal remains far off’ – That this is controversial and not supported by all experts is a main message – ‘Publicized by his university with a sensationalized sell line’
Singapore	1/1	– Careful in drawing conclusions
UK	2/2	– Warns experiments are difficult to perform and can be misinterpreted – ‘While there is good evidence that epigenetic inheritance happens in plants and worms, mammals have very different biology. Surani's lab carried out thorough studies on how epigenetic information was erased in developing mouse embryos and found that “surprisingly little gets through” the reprogramming process’ – ‘Prof Timothy Bestor, a geneticist at Columbia, is far more damning, claiming that the entire field has been grossly overhyped. “It's an extremely fashionable topic right now. It's very easy to get studies on transgenerational epigenetic inheritance published”’ – ‘Work was “poorly presented”, with a lack of detail that makes it difficult to interpret the results. Bestor adds that they failed to explain how a response associated with the nose managed to pass all the way into sperm. Like all other epigenetic inheritance studies, he says: “There is a total lack of plausible mechanism”’ – ‘Surani says that researchers who are not getting positive results are finding their work more difficult to publish, which is feeding hype around the field. “There have been people who have tried to replicate some of this stuff and it doesn't work,” he says’ – ‘This is a pilot study on a small number of special patients so we're not making any generalized statements on the causes of autism’, said Professor Andrew Feinberg of Johns Hopkins University
Hong Kong	1/2	– ‘But there are controversies, notably regarding a study that found pesticide and fungicide impacts on pregnant rats led to changes that persisted for at least four generations—results that have proven hard to replicate’ – ‘There are some doubts, too, regarding a study of people in Överkalix’ – ‘As yet, the results are based on a small sample size, and it is far from certain that the aggression is rooted in epigenetics’
Japan	1/2	– Balanced article about Roadmap project
USA	2/5	– ‘Could the same effect be shown to be taking place in humans?’ – ‘This was found in a mouse model, not a human, so it might not apply to us. But if it does, it could give researchers new areas to target when they try to treat the disease’ – ‘But while the researchers are confident that their discoveries will be revelatory, they also see a long road ahead. They will find circuits, another author, Anshul Kundaje, an assistant professor of genetics at Stanford said. But, he added: “Making sense of them is a whole different story”’
Germany	0/2	– States the result is ‘Not only in animal models’

The ‘balance’ score is based on the inclusion of “moderating” views or evidence in the news stories, which are presented here.

These are just a few examples of balanced reports, which in many cases stem from the inclusion of interviews with epigenetics researchers who were not involved in the original studies, such as this section from a feature article in the UK press:

‘Professor Timothy Bestor, a geneticist at Columbia University in New York, is far more damning, claiming that the entire field has been grossly overhyped. “It's an extremely fashionable topic right now. It's very easy to get studies on transgenerational epigenetic inheritance published”’. [28]

The situation was similar in the reporting 5 years later. Several news reports failed to present moderating views of epigenetic science or an indication of epigenetics research limitations (4/10), and did not include anything more than a statement that ‘the science of epigenetics is still emerging’ or ‘further research is necessary’.

We note that while a news story may be balanced in this sense of reporting caveats and alternative viewpoints, the studies in question as well as the language used (especially if hyped) will have an impact on how readers engage with the evidence. The research reported throughout the articles involved emotional topics, including studies of pregnant 9/11 survivors, of women pregnant during the 1998 ice storm in Quebec, and of children of Holocaust survivors.

Case Study: Evidence Supporting Messages about Parental Responsibility

To further understand the distortions we observed in the press, we conducted a case study of the reporting of scientific evidence in the five news stories from 2014 to 2015 that emphasized the policy theme ‘parental responsibility’, which included bold statements such as:

*‘*Only mothers have the power to change the evolution of obesity’ [21].

‘…suggest a vicious multigenerational cycle’; the new studies show that maltreatment, more prevalent in poor families, ‘damages children and perhaps even their children’s children at the most fundamental biological level’ [29].

‘Parents could suddenly find themselves responsible for passing on not only their poor genes, but also their poor lifestyles’ [28].

**Figure 1:**
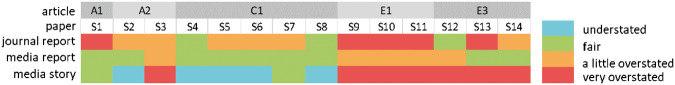
heatmap showing “hype” in reports of research results in scientific publications (journal report), media descriptions of the research (media report), and in the context of media stories (media story)

We traced the supporting evidence referred to in these news stories to the scientific publications directly reporting the research. We then assessed the communication of the scientific research at three different stages: in the academic scientific journal publication, in the media description of the scientific results per se [the news story text describing the research finding(s) in isolation], and finally, in the overall media account of the research [within the broader context of the news story]. This analysis was carried out for a total of 14 scientific research studies that were referred to by the press. For the academic publications, the main causes of “over-statement” were noted (see Table 4). Our aim was to better understand how any distortions and overstatements regarding epigenetic research could have arisen. Often, three (*n* = 6) or four (*n* = 3) separate issues were noted per scientific report. Figure 1 shows that 11/14 scientific reports were either ‘a little’ (*n* = 6) or ‘very’ (*n* = 5) overstated (scored on a four-point scale: ‘understated’, ‘fair’, ‘a little overstated’, and ‘very overstated’).

**Table 4:** research issues with the scientific studies reported on, which are likely to affect reported research outcomes, and their occurrence (*n*) amongst the scientific publications (see case study)

Research issues	(*n*)
Transient gene activities, not epigenetic effects	3
Samples (cell type)	9
Coverage (no. genes)	2
Sample size	11
Control group (size and quality)	1
Contorted argument to support hypothesis	2
Difficulties studying humans (control for environment)	6
Statements not supported by references provided	1
Extension to human difficult (limitations of experimental model)	2

Several issues affecting the strength of the reported outcomes of the research were common to other areas of genetics: limitations due to sample size and reporting “significance” even if a large number of hypotheses were tested and no more than the expected number of differences were seen. Of particular note is the general difficulty in partitioning nature from nurture in studying human conditions—labeling differences in disease occurrence and/or specific biochemical measurements as “epigenetic inheritance”, where even genuine, validated differences could also be driven by shared genetics or shared current living environment. Similarly, some studies relied on a specific molecular hypothesis stemming from animal studies. However, in transition from model organism to human study the experimental design is compromised with respect to the tissues studied as well as functional differences in genomes across species, rendering the animal model hypothesis untestable in human. Finally, generally small samples in human studies are compounded by lack of resources to independently replicate reported findings. Furthermore, some cohorts rely on rare historic conditions or contemporary events that are unlikely to reoccur.

Along with the “hype” we observed in scientific reports, the heatmap in Fig. 1 shows the four-point scale scores for both the media descriptions of the scientific research and the overall media stories. Media descriptions of the reported research (in isolation) ‘downplayed’ the overstatement seen in the academic publications in all but two cases (Supplementary S3 and S12). The broader media narrative either continued to tone down the research results reported in the scientific literature (*n* = 7), or conversely, hyped them within the overall story (*n* = 7). With a single exception (Supplementary S12), when hyping occurred in the media, the original scientific publication already showed a degree of overstatement. A previous study of media reporting of genetic discoveries and associated technology has also shown much less hyping by the media than is commonly assumed [30].

This case study demonstrates that epigenetic research results are often overstated in scientific reports as well as mainstream news reports, potentially contributing to some of the hype seen in the media.

### Science Communication Challenges

Experimental limitations and communication challenges in epigenetics research have previously been discussed by leaders in the field [31–34], with the Berlin-Brandenburg Academy of Sciences and Humanities recommending: ‘An objective and critical dialogue about epigenetic topics in the sciences and with the general public should be promoted to a greater degree in order to arrive at a more differentiated estimation of the importance of epigenetics which goes beyond natural scientific aspects and examines social issues’ [35]. Interviews with epigeneticists in several countries have further documented scientists’ concerns about science communication challenges in the field, including the hyping of scientific results and controversy regarding the plausibility of transgenerational epigenetic inheritance [36, 37].

The excitement afforded by new ways of measuring epigenetic variation in genomes is reminiscent of the early days of genetic association studies, where insufficient replication and small sample sizes often led to reporting of biologically tantalizing associations that failed to hold true in later studies [38]. The statistical guidelines that have been developed to safeguard against over-reporting genome-wide association study (GWAS) findings are complex to adapt to epigenetics as over-reporting of results may be related to many unknown random and systematic confounders in sampling and measuring, which may just be repeated in follow-up studies. Consequently, as opposed to hypothesis-free GWAS, the scientific community and publishers need to collectively define reasonable criteria by which to evaluate epigenetic studies, which—despite the epigenome-wide nature of our technological abilities—will likely require some examination of plausibility of hypothesis, appropriateness of study design, and follow-up validation study of mechanistic action. Such standards, which will undoubtedly limit areas where human epigenetic phenomena may be credibly studied, will be key to resolving public misconceptions and helping popular media differentiate data from speculation. Second, while the addition of new genomic measures (e.g. methylation reflecting regulatory element change) can be confirmatory for earlier reported gene expression effects, transient phenomena (demonstrating little “cellular” or “epigenetic” memory) observed in cells undergoing metabolic stress and remodeling should not be labeled as an epigenetic phenomenon that would somehow alter the long-term function and fate of the respective organ system.

The IHEC Bioethics Workgroup recently proposed Points-to-Consider on the Return of Epigenetic Research Results to guide decisions about returning epigenetic research results to research participants (so-called return of research results) (available at http://ihec-epigenomes.org/fileadmin/user_upload/documents/Points-to-Consider_on_the_Return_of_Epigenetic_Research_Results.pdf, accessed 19 Aug 2020) [39]. This Points-to-Consider framework includes criteria for classifying the level of evidence of epigenetic findings as either Associated, Inferred, or Causal Variants. Associated Variants are supported by statistics only (e.g. in an epigenome-wide association study). Inferred Variants are supported by statistics and inferred functional evidence (e.g. involvement in plausible mechanism inferred from additional data). And finally, Causal Variants are variants for which disease-causality has been demonstrated. Similarly, we can point to the difference between ‘statistical significance’ (often easily reached), ‘biological significance’ [in this context, any cellular effect conferred by epigenetic change(s) due to either changes in cell composition rather than gene regulation, or very low methylation differences, for example], and ‘significance for an individual’s health or life, or transgenerational effects’. Such classifications may be helpful in communicating the strength of evidence and potential implications of epigenetic findings. Indeed, a recent study of epigenetics knowledge claims in public discourse found them to be mostly correlative and easily overextended toward causation [20].

To further address these science reporting and communication challenges, we prepared a Press Kit template that epigenetics researchers can use to provide colleagues at institutional press offices with relevant and accurate information about new epigenetic research findings, including descriptions of common caveats and of ways in which new research findings might be mis- or over-interpreted (see Supplementary materials). The Press Kit resulted from discussions with members of a social epigenetics laboratory who expressed concerns about some of the media and social media coverage of publications by the group that touched on aspects of parental care, maternal environment, and related early life issues. It was designed to improve stages of the science communication process that the team could directly influence by providing a cautious and balanced description and interpretation of laboratory findings to the university press office. It has been used by this research group several times and received positive feedback from the researchers who used it, and also from the university’s press office. This approach is supported by the results of a recent trial which showed that press releases that included statements of caution regarding the interpretation of research findings (explicit causality statements and caveats) led to an increase in media reporting of study limitations, and this without any observed decrease in news uptake [40]. Our Press Kit template would help provide media and communications professionals with easy access to the information they need to create institutional press releases that accurately reflect the significance as well as limitations of individual research publications and of epigenetics as a field.

## Conclusion

Epigenetics is a burgeoning and very exciting field of investigation. Further evaluation of evidence of epigenetic harm and benefit in many of the areas discussed in mainstream media coverage (e.g. diet, exercise) will be important to enable both public understanding and robust clinical translation of this science. Our analysis highlighted a tendency for both scientists and journalists to overstate epigenetic research results. It also revealed several recurring themes in the press coverage of epigenetics-related news stories, including an emphasis on its immediate consequences for individual and collective responsibilities. Left unchecked, this could lead to greater public concern than may be warranted by current scientific advances in the field. Such misrepresentation could also impact the public understanding of epigenetics and epigenetic risk in the long-term, potentially hindering future healthcare applications of the science and leading to ill-suited policy.

## Supplementary data

Supplementary data are available at *EnvEpig* online.

## Supplementary Material

dvaa015_Supplementary_Data
