# Supplementary material for: Communicating science: epigenetics in the spotlight
Source: Environ Epigenet. 2020 Nov 18;6(1):dvaa015. doi: 10.1093/eep/dvaa015 (PMC7673471; doi:10.1093/eep/dvaa015)
Supplement: dvaa015_Supplementary_Data [file dvaa015_supplementary_data.docx]

**Supplementary Material**

Press Kit for Researchers and Press Offices

Information to provide about your epigenetics research study (with example text):

**Title:**

**Authors:**

**Senior/corresponding author and affiliation:**

**Main local contact(s), if different to above:**

**Social media handles of main local author(s):**

**Journal:**

**Publication date:**

**Embargo details:**

**Primary goal of our study**

- [EXAMPLE] to develop improved diagnostic biomarkers for [condition]

**Problem(s) we were trying to solve**

- [EXAMPLE] earlier diagnosis of [condition] can help people access appropriate care and services, and can improve outcomes – but at present there is no definitive diagnostic test for diagnosis of the early stages of the disorder

**How does this study build on the lab’s previous work on the same topic?**

- [EXAMPLE] we have previously worked with rodent models of [condition], and found distinctive epigenetic patterns during the earliest stages of the disorder. The current study has validated and expanded on these initial findings.

**What we did**

**Our results**

**Key conclusions**

**Did any of the results surprise you? Why?**

**Who can use these results, and how? (include short- and long-term applications, if possible)**

- [EXAMPLE] short-term – other researchers can look for the same epigenetic signature in their own data, which will help to determine if this is a general phenomenon and provide insights into the prevalence of [condition] in different populations
- [EXAMPLE] long term – if this biomarker is validated, physicians can potentially use it as an additional diagnostic and care planning tool for people with suspected [condition]

**Why will members of the public be interested in these findings?**

**What we plan to do next**

**Caveats – what are the study’s limitations, and what does it *not* tell us? How might it be over- or mis-interpreted by non-experts?**

- [EXAMPLE] small and/or preliminary study; animal or cell line model
- [EXAMPLE] we found a *correlation* between the disorder and the epigenetic mark – we do not know yet what causes the epigenetic change, or how/whether it affects the health of any individual
- [EXAMPLE] there is a danger that this study could be used to increase individual or parental blaming / shaming
- [EXAMPLE] people could potentially try to use this study to market nutritional supplements or other “alternative” interventions that are often claimed to alter epigenetic marks and “clean dirty genes”
